# Genetic analysis of Indian tasar silkmoth (*Antheraea mylitta*) populations

**DOI:** 10.1038/srep15728

**Published:** 2015-10-29

**Authors:** Saikat Chakraborty, M Muthulakshmi, Deena Vardhini, P Jayaprakash, J Nagaraju, K. P. Arunkumar

**Affiliations:** 1 Centre of Excellence for Genetics and Genomics of Silkmoths, Laboratory of Molecular Genetics, Centre for DNA Fingerprinting and Diagnostics, Tuljaguda, Nampally, Hyderabad 500001, India; 2 Centre for Ecological Sciences, Indian Institute of Science, Bangalore 560012, India; 3 Muga silkworm seed organization and Eri silkworm seed organization, Central Silk Board, Guwahati 781006, India

## Abstract

Indian tasar silkmoth, *Antheraea mylitta* is an economically important wild silkmoth species distributed across India. A number of morphologically and ethologically well-defined ecotypes are known for this species that differ in their primary food plant specificity. Most of these ecotypes do not interbreed in nature, but are able to produce offspring under captive conditions. Microsatellite markers were developed for *A. mylitta*, and out of these, ten well-behaved microsatellite loci were used to analyze the population structure of different ecoraces. A total of 154 individual moths belonging to eight different ecoraces, were screened at each locus. Hierarchical analysis of population structure using Analysis of MOlecular VAriance (AMOVA) revealed significant structuring (F_ST_ = 0.154) and considerable inbreeding (F_IS_ = 0.505). A significant isolation by distance was also observed. The number of possible population clusters was investigated using distance method, Bayesian algorithm and self organization maps (SOM). The first two methods revealed two distinct clusters, whereas the SOM showed the different ecoraces not to be clearly differentiated. These results suggest that although there is a large degree of phenotypic variation among the different ecoraces of *A. mylitta*, genetically they are not very different, and the phenotypic differences may largely be a result of their respective ecology.

Populations of several species are further classified by taxonomists into subspecies, races, demes, clines and so on, of which only cline and deme have non-arbitrary definitions^1^. Ecotypes consist of genetically distinct subsets of populations in a species, that are specialized to particular environments^2^,^3^. More generally, ecotypes can be defined as subsets of populations within a species that have different fundamental niches^4^. The Indian tasar silkworm, *Antheraea mylitta* is a natural fauna of tropical India, distributed in different geographical locations and habitats in this country. Possibly, because of the distinct ecological conditions prevailing in these different localities, several morphological variants, traditionally called ecoraces, have been identified in *A. mylitta*^5^. As high as 44 ecoraces are reported in this species, which feed primarily on *Terminalia* (Family: Combretaceae) species and *Shorea robusta* (Family: Dipterocarpaceae), and also on a number of secondary food plants^5^. The genus *Terminalia* and *Shorea* are quite far phylogenetically. Although both are Rosids, *Terminalia* belong to order Myrtales and *Shorea* to Malvales^6^. The ecoraces are uni, bi or trivoltine depending upon the geo-ecological conditions and differ from each other in several qualitative and quantitative traits^7^, such as cocoon weight and colour, larval colour, and so on. Although most of these ecotypes do not interbreed in nature, some of them produce offspring when mated in captivity^8^. Tasar cocoons are reported to be the largest among all the silk-producing insects in the world^9^. The silk fibre produced by *A. mylitta* has its own distinctive colour, and is coarser than *Bombyx mori* silk. However, the tasar silk has higher tensile strength, elongation, and stress-relaxation values than the silk secreted by the domesticated silkworm *B. mori.* These properties have made tasar silk as competent and desirable as *B. mori* silk^10^,^11^.

Since, *A. mylitta* primarily inhabits forested habitats, it is expected that with the gradual depletion of forest cover due to surge in the human activities the habitat lost its continuity and resulted in geographic isolation. This isolation might have allowed the populations to continue separately for generations, resulting in different ecoraces. There is quite a bit of ambiguity in naming of these ecoraces. As the boundaries between the ecoraces are often fuzzy and the races do not obey the concept of static boundaries, the racial identity seems to be arbitrary at times. Quite surprisingly, two crops of the same population are sometimes named as two different ecoraces^12^.

There is a lack of well-characterized molecular markers to study *A. mylitta* ecoraces. Although few studies have been carried out with RFLP^13^, RAPD^14^, SCAR^15^, ISSR^12^,^16^ and other DNA markers^17^, except for RFLP, the rest are dominant markers, and hence the estimation of allele frequencies are based on the assumption that the loci are in Hardy-Weinberg equilibrium. Also, barring a couple of reports on *Antheraea assama*^18^,^19^,^20^, there are no reported studies that describe the detailed population genetics of saturniid silkmoths, using SSR markers.

The pronounced phenotypic and behavioral variation of *A. mylitta* ecoraces have made it difficult for researchers to identify ecorace specific phenotypic markers^8^. Therefore, there is a need to identify genetic markers for a specific phenotype to differentiate ecoraces. Are these ecoraces genetically distinct from each other? Do the ecoraces form structured population? Are any of these ecoraces in decline? These questions are of considerable importance to the biology of *A. mylitta*. With these points in mind, we developed 32 microsatellite markers and screened eight *A. mylitta* ecoraces collected from different geographical locations across India to obtain insights into the population genetics of these ecoraces (Fig. 1 and Table 1).

## Results

### Genetic variation

#### Descriptive statistics

Considerable variation was observed at all microsatellite loci. Except Bhandara, which was monomorphic at the locus Amysat013, all other populations showed polymorphism at all the 10 loci (Supplementary data 1). Number of alleles ranged from 1 (Locus: Amysat013, Population: Bhandara) to 15 (Locus: Amysat023, Population: Modal) across the ten microsatellite loci studied among the eight ecoraces. The average number of alleles per locus ranged between 2.5 ± 0.76SD (Amysat013) and 11.75 ± 2.05SD (Amysat023). The minimum average number of alleles for a population across all 10 loci was 3.7 ± 1.77SD for Sukinda and the maximum was 6.6 ± 4.24SD for Modal. Average observed heterozygosity per locus (H_o_) taking all the populations together ranged from 0 (Amysat013) to 0.81 ± 0.12 (Amysat023). The minimum average H_o_ across all the loci was observed in the population Daba Trivoltine (0.25 ± 0.32) and maximum in Sukinda (0.37 ± 0.34).

#### HWE

Hardy-Weinberg exact tests were done for all the ten loci and inferences were drawn based on the sequential Bonferroni corrected α (Supplementary data 2, Table 2). No test could be performed for locus Amysat013 for the Bhandara population, as the locus was monomorphic in this population. Also GENEPOP did not return any result for the locus Amysat037 in case of the populations Modal and Daba Bivoltine. Closer inspection of the data revealed that in these two populations, the expected (H_e_) and observed heterozygosities (H_o_) for this locus are exactly the same. Generally, the more similar H_e_ and H_o_ are, the higher is the probability that the locus is in HWE. This is supported by the observations in all other loci. A similar exact test^21^,^22^ was also performed in ARLEQUIN 3.0^23^, where the p-value of the HWE test in both these cases was 1.0. Hence, these two scenarios were inferred with a p-value of 1. Six of the ten loci were in HWE in at least two populations. Amysat026 was in equilibrium in all the eight populations and Amysat001 was in equilibrium in seven populations. Both Amysat023 and Amysat037 were in equilibrium in six populations. Only Amysat013 was not in equilibrium in any of the populations. Seven out of ten loci were in equilibrium for the Sukinda population, and for Sarihana, only two of the loci were in equilibrium. Among other reasons, departures from HWE expectations may occur in a case where a locus contains null alleles. MICROCHECKER was used to evaluate the effect of null alleles on the results. Except four loci (Amysat001, Amysat023, Amysat026, Amysat037), all other loci were observed to have significant homozygote excess indicating the presence of possible null alleles.

#### LD

Exact test for genotypic disequilibrium was performed using Markov chain simulations to identify significant association between pairs of loci. Tests for each population as well as global tests were carried out. As there are 10 loci in all, there can be 45 possible pairs. However, due to lack of polymorphism for Amysat013 in the Bhandara population, only 36 tests could be performed for this population. Out of these 351 total comparisons, significant LD, following sequential Bonferroni correction, could be observed in only three cases, i.e. Loci Amysat023 & Amysat032, Amysat026 & Amysat032 and Amysat023 & Amysat026, all in Sukinda population. In case of the global test, following sequential Bonferroni correction, only three pairs of loci (Amysat023 & Amysat032, Amysat023 & Amysat026 and Amysat026 & Amysat032), out of 45 comparisons were found to be under LD. All other loci segregated independently of each other (Supplementary data 3).

#### F-statistics and AMOVA

The locus specific fixation indices are shown in Table 2. Except Amysat026 and Amysat032, all other loci had a significant F_ST_ value. F_IS_ values were significant for all loci, except Amysat026. Result of the AMOVA, along with the fixation indices and their bootstrap confidence intervals are shown in Table 3. As the proportion of missing data was very less, the results of the haplotype as well as the locus by locus analysis were very similar. Here, results of only the locus by locus analysis are reported. Weighted average of the locus by locus F_IS_ value was considerably high (0.505), suggesting significant inbreeding within these populations. F_ST_ was also observed to be significant (0.154). In accordance with these values, 15.4% of the covariance was assigned to the across populations and 43% to the within population component. About 42% of the covariance could be assigned to the within individuals component.

#### Mantel test

The samples for Daba Bivoltine and Daba Trivoltine were collected from the same locality and hence geographic distance between them was zero. The maximum pairwise physical distance, i.e., 1034.4 km was between Sarihana and Andhra Local populations. Pairwise F_ST_ values ranged from 0.015 (between Sarihana and Daba Bivoltine populations) to 0.339 (between Bhandara and Sukinda populations). All pairwise genetic distance values were significant except the one between Sarihana and Daba Bivoltine populations (F_ST_ = 0.015, p = 0.44) (see Supplementary data 4). The Mantel test revealed significant positive correlation between genetic and physical distance r = 0.59, p = 0.026 (Fig. 2). The Mantel test between the residuals of a km-F_ST_ regression, however, was found not to be significant (r = 0.35, p = 0.055) suggesting a lack of migration-drift equilibrium (Fig. 3).

#### Bottleneck analysis

In the BOTTLENECK analysis, none of the populations showed significant excess of simulated gene diversity compared to the observed gene diversity either under IAM or TPM, suggesting lack of recent bottleneck in any of these eight populations.

#### Population analysis using distance methods

The NJ tree constructed based on Nei *et al.*’s D_A_ distance, showed two major clusters (Figure S1), one with Andhra Local and Bhandara populations, and another included remaining six ecoraces (Raily, Modal, Sukinda, Sarihana, Daba Bivoltine and Daba Trivoltine). NJ tree did not show any specific relation among these six ecoraces. Although the two Daba ecoraces clustered together, the bootstrap value was found to be too less to make any strong conclusion.

#### Bayesian analysis of probable population clusters

The results of STRUCTURE analysis were very similar to that of the phylogenetic analysis. The modal value of ΔK was found to be at K = 2, suggesting that the data is best explained consisting of only two populations. One group containing two ecoraces (Andhra Local and Bhandara) of southern and central India and the second group consisted of remaining six ecoraces (Raily, Modal, Sarihana, Sukinda, Daba Bivoltine and Daba Trivoltine) mainly from eastern India (Fig. 4).

#### Artificial neural network

The SOM output layer consisted of a 11 × 6 grid (Fig. 5). The map had a topographical error of 0. The quantization error was also reasonably small (1.792) suggesting the map quality to be good. The distances between neighbouring units on this map are shown in Fig. 6a. One can also visually inspect the level of clustering by comparing Fig. 6a and Figure S3. A better visualization can be obtained by looking at the hit histograms (Figure S2 in Supplementary file). From Fig. 5b, it appears that ecoraces Andhra Local (red) and Bhandara (green) are clustered together. However, this is a crude way of clustering the data as boundaries of each cluster are difficult to visualize in this way. Therefore, this SOM was further subjected to cluster analysis using Ward’s linkage (minimum variance criterion). The resulting dendrogram is shown in Fig. 6 (A higher resolution version can be found in Supplementary file, Figure S4). The Davies-Boulding clustering index^24^ indicated the best clustering to be 7 (Minimum value of the index 0.954 at level 7). The cluster boundaries are also indicated in Figure S3. If a cut-off is taken at squared Euclidean distance of 5, Group 1 and the rest forms two distinct clusters, Group 1 consist of the highest number of neurons among these 7 groups and is dominated by Andhra Local and Bhandara populations. However, these two ecoraces are not exclusive to this group and can be found in other groups as well.

## Discussion

In this paper, we have characterized the microsatellites of *A. mylitta* and used them to study the genetic structure of its different ecoraces. Like most other species studied, we observed dinucleotide microsatellites to be the most abundant, followed by tri- and tetra nucleotide. Among the dinucleotide microsatellites, those with (CA) motifs were the most abundant. CA/GT repeats are generally the most common dinucleotide repeat in a wide variety of vertebrates and arthropods^25^,^26^. Insects^27^,^28^, including lepidopterans^29^,^30^,^31^,^32^,^33^,^34^,^35^,^36^,^37^ are no exception.

There have been studies investigating the nature and distribution of genetic variation in wild lepidopterans. However, these have mainly focused on threatened or declining species^38^,^39^,^40^ or pests^41^,^42^,^43^,^44^. Among other silkworms the genetic structure of *A. assama*, a species with a very restricted distribution, has been studied and its populations were found to be reasonably differentiated^18^,^20^. Different strains and lines of the domesticated silkworm *B. mori* have also been subjected to this kind of analysis^32^,^45^,^46^,^47^, however these studies are arguably not comparable to wild lepidopteran species.

A couple of studies using ISSR markers to analyze the intra-race diversity in Raily^16^ and Daba^12^ ecoraces of *A. mylitta* revealed considerable genetic differentiation across and within the populations of both the ecoraces. In another work, using RAPD markers, genetic variation among the different ecoraces of this moth were assessed^14^. However, none of the previous works did a detailed study of the genetic structure of the different *A. mylitta* ecoraces.

Here, we have studied the genetic structure of *A. mylitta* for a large part of its distribution. Eight ecoraces distributed as far as 1000 km apart were collected and their population genetics was investigated using ten polymorphic microsatellite loci. There was significant deviation from HWE in some of the loci in a few populations. This can be because of several reasons- 1) There was an overall excess of homozygotes than one would expect just by chance alone as reflected by the high locus specific F_IS_ values as well as its weighted average (0.505) across loci in the AMOVA (Table 3). As has been reported in the ‘Methods’ section, the sample cocoons were collected within about 500 m radius of forest area, and may consist of offspring of the same mother. This may be a possible reason of observing such a high F_IS_ value. 2) The sample sizes for each population was consistently low (Maximum N = 22, Minimum N = 15), and may not represent every genotype adequately, and 3) Presence of null alleles, as was found using MICROCHECKER, which in turn may result in the excess of homozygote in all the loci. Significant association was absent in almost all locus pairs in all the populations. Even globally, i.e., when all the populations were included together, there was significant association between only three of the possible 45 pairs of loci.

AMOVA revealed significant structuring of *A. mylitta* populations in the region under study (F_ST_ = 0.154). Although result of the Mantel test was significant (r = 0.59, p = 0.026), there was an absence of migration-drift equilibrium as reflected by the lack of significant correlation between the residuals of a km-F_ST_ regression and km (r = 0.35, p = 0.055). Excluding the localities with non-significant pairwise F_ST_ values, i.e., Daba Bivoltine and Sarihana, either sequentially or together, did not change this pattern, i.e., the km-F_ST_ correlation was consistently significant, but the km-residual Mantel was not significant. This suggests a lack of migration-drift equilibrium in the region studied. One may posit from the extent of scatter of individual points in Figs 2 and 3 that random genetic drift has been relatively more important than migration in shaping the current genetic structure of *A. mylitta*. However, as there are not too many points in the short distance range, this lack of equilibrium can also be an artifact of the sampling regime^48^.

Both distance method and Bayesian analysis divided *A. mylitta* populations into two groups. One group consisted of one population each from southern and central India (Andhra Local and Bhandara). Another consisted of populations from eastern India. To investigate this pattern further, Andhra Local and Bhandara was taken as one group and the rest of the six populations were taken as another and an AMOVA was performed, giving 17% of the covariance assigned to the “Among groups” component and 5% to the “Among populations within groups” component, as compared to the overall “among populations” component of 15.41%, suggesting the possibility of substructuring. This is in full agreement with the STRUCTURE analysis. Remarkably, the two Daba ecoraces, though occurring sympatrically was found to be genetically distinct (F_ST_ = 0.06452). This is in line with the previous report^14^, wherein they have used RAPD markers to differentiate 10 ecoraces of *A. mylitta*. In the NJ tree (Figure S1), except for the cluster consisting of Andhra Local and Bhandara ecoraces, all other bootstrap values were quite low (33 to 53). We further investigated this pattern using Kohonen maps. Although the Bayesian clustering algorithm and SOM have been shown to behave similarly for multilocus genotype data^49^, in the present study, the results of the two methods were not exactly the same. A hit map of the eight ecoraces revealed samples from Andhra Local and Bhandara sharing the same or nearby neurons (Fig. 5b and Supplementary Figure S3). However in the hierarchical cluster analysis the pattern observed was not as clear as that of the phylogenetic and Bayesian analysis. Although many samples from Andhra Local and Bhandara ecoraces did cluster together [Cluster 1 (Group I) in Fig. 6], there were other clusters in which other samples from these ecoraces were distributed (Fig. 5b and Supplementary Figure S3).

**Figure 1:**
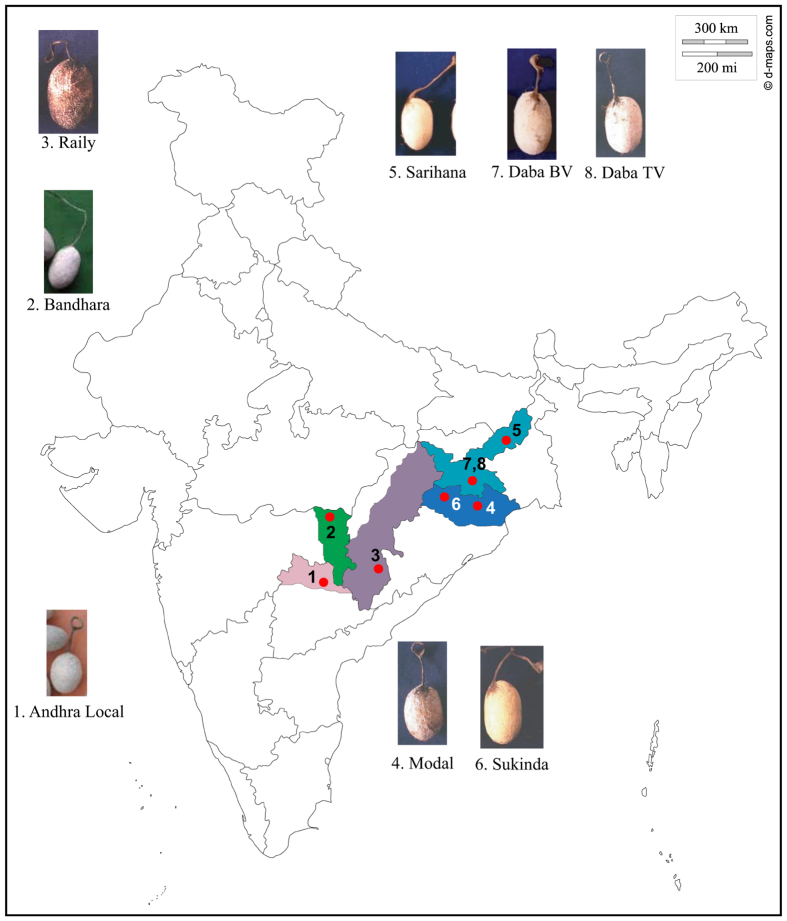
Spatial distribution of *A. mylitta* ecoraces used in this study. The map template was downloaded from d-maps.com^109^ and the distribution of the different ecoraces were defined upon it using Adobe® Photoshop®. The photographs of the cocoons were taken by one of the authors, Dr. P. Jayaprakash, which were then included in the figure using Adobe® Illustrator®.

**Figure 2:**
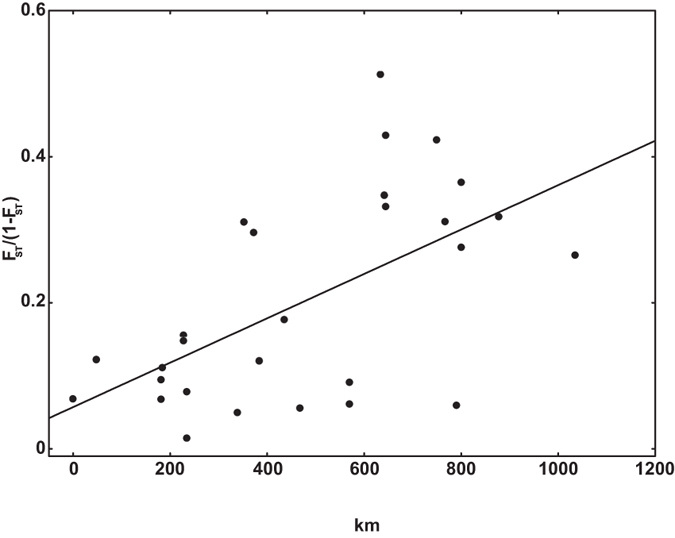
Relation between physical distance and genetic distance. The regression line represents the equation *F*_*ST*_ */(1 F*_*ST*_ ) = 0.0003*km + 0.0571, p < 0.001; R^2^ = 0.35. Mantel test r = 0.59, p = 0.026.

**Figure 3:**
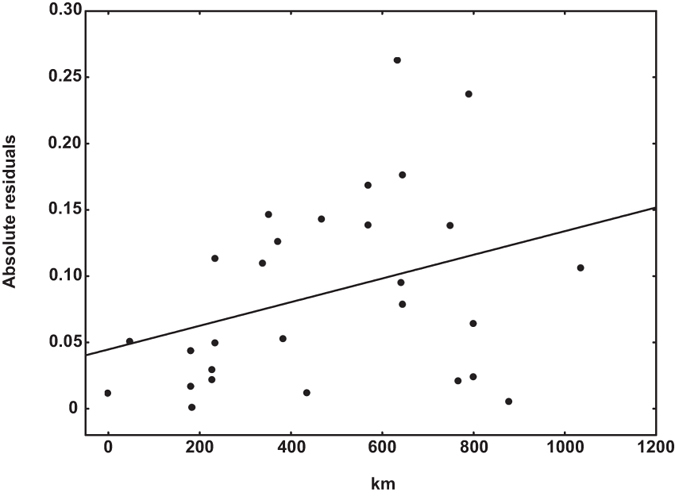
Relation between the residuals of the regression in Fig. 2 and physical distance. The regression line represents the equation Residuals = 8.9251E-5 *km + 0.0447, p = 0.07; R^2^ = 0.12. Mantel test r = 0.35, p = 0.055.

**Figure 4:**
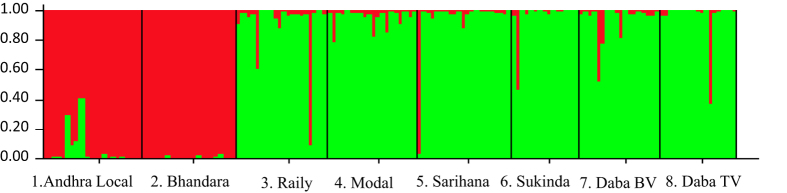
Population structure grouped by *A. mylitta* ecoraces using STRUCTURE at K = 2.

**Figure 5:**
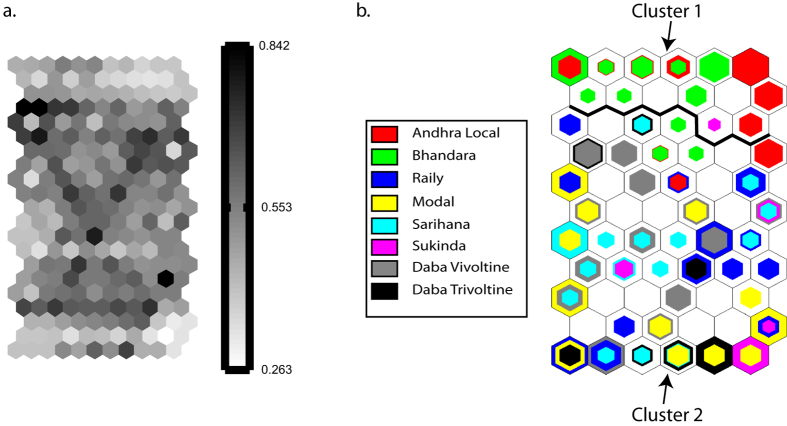
Self organization map. The u-matrix is shown in Fig. 5(**a**). The color bar represents distance between neighbouring neurons. The smaller the distance the closer are the neurons from each other. The respective individual labels on this map are shown in Figure S3. **(b**) shows the same map with different colour for each ecorace. The two main clusters obtained through cluster analysis are delineated with a solid black line.

**Figure 6:**
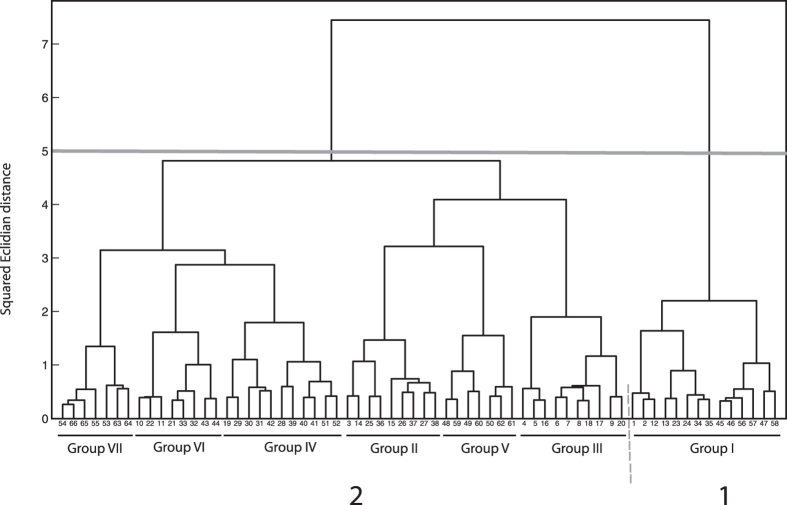
Dendrogram obtained following hierarchical cluster analysis of the SOM. The numbers on the tip of the trees are the same neuron numbers shown in Figure S3. Seven distinct clusters could be identified based on Davies-Boudin index. These have been labeled as Group I to VII. Moreover, taking a cut-off at squared Euclidian distance of 5 two main groups, 1 and 2 could be identified. A comparison of this dendrogram with Fig. 5b and Figure S3 would suggest that cluster 1 consisting only of Group I neurons is represented almost exclusively by individuals from Andhra Local and Daba ecoraces. (See text for more details).

Clustering of Andhra Local and Bhandara has also been shown previously using RAPD markers^14^. Andhra Local and Bhandara have adjacent distributions with no obvious geographic barrier between these two and the other six ecoraces (Fig. 1). This relationship shown in the phylogenetic tree is in line with its distribution geographically.

The *A. mylitta* ecoraces have considerably different niches. They have different host plants, are behaviourally different, have different cocoon colours and commercial characteristics^17^. The differences are so marked that they are popular with their unique local names in particular regions. Though there is a notion that each ecotype is specific to a particular region, overlapping distribution across the contact zones cannot be ruled out^50^. Experiments carried out to evaluate the effect of different food plants on cocoon characteristics revealed that food plants also have a considerable effect on these characteristics^51^.

Though different morphologically identifiable ecoraces are found in different geographical regions of India, we speculate that these may be the result of a particular ecological condition prevailing in that region, and not because of their distinct genotype. Considering the lack of ecorace specific structure observed in this study, we opine that there may be less number of genetically distinct ecoraces than reported earlier. There seems to be a greater influence of environment such as host plants, climatic conditions, geographical locations etc., in shaping the phenotype of these ecoraces. Therefore, it is likely that most of these ecoraces are genetically similar but differ in certain morphology. *A. mylitta* is a highly polyphagous species, and displays a wide range of adaptation. Lepidopteran populations are known to be affected by habitat quality^52^ and food plants^53^. Either or both of these factors could be responsible for the variation in phenotype observed among these ecoraces.

In this current work, we present a detailed genetic study of eight different ecoraces of a wild silkworm *A. mylitta*. However, one should keep in mind that we do not have replicates of the ecoraces in our dataset, i.e., each ecorace has been sampled only from a single locality and hence the effect of ecorace and locality might be confounded in the pattern we have observed.

Future work should concentrate on doing a more detailed behavioural and ecological study to understand the natural history of *A. mylitta* in greater detail. Cross feeding different ecoraces with the primary food plants of each other will help in confirming or ruling out the nature of host plant as a cause of phenotypic variation. Other ecological factors, similarly, can be tested, either sequentially or together, to get into the root cause of the origin of these ecoraces. Recent developments in statistical modeling such as the generalized linear model and the suit of different mixed models, allows one to carry out such investigations with great efficiency and cogency. Moreover, performing rigorous mating experiments may help us to understand the cause and nature of reproductive barrier among the different ecoraces. From the perspective of its genetics, identifying the hybrid zones of different ecoraces and studying the population structure including those regions may give a finer understanding of their population genetics. Development of additional microsatellite markers and employing a more extensive sampling regime including samples at a finer distance scale would improve the resolution of the understood population genetic structure and, intra- and inter-race diversity of this species.

## Methods

### Collection of samples of different ecoraces

*A. mylitta* is an exceptionally versatile species as its ecoraces vary, in terms of phenotype, voltinism, distribution and also in their choice of food plants, and some of the ecoraces do not freely interbreed^54^. For example, the ecoraces Andhra Local, Bhandara and Sarihana are notable for their smaller cocoons than the others. On the other hand, the ecoraces Raily and Modal produce silk with significantly longer filament length than the other ecoraces. Wild populations of Daba can be both bi and trivoltine in nature, while semi-domestic counterparts are exclusively either bivoltine or trivoltine. Ecoraces Modal and Raily are wild in nature and are found exclusively in natural habitats^12^. The cocoon characteristics, sampling locations and information on primary food plants of all the eight ecoraces (Fig. 1) reported in the presented study are given in Table 1.

Live pupae were collected from their natural habitats in eight geographic locations representing eight ecoraces (Table 1 and Fig. 1). At each sampling site, wild cocoons were collected from the respective host plants. All sampling at each site was done within ~500 m radius of forest area.

### Construction of repeat enriched genomic library and sequencing

For the construction of genomic library, DNA was isolated from *A. mylitta* pupae using the method described earlier^55^. Genomic DNA was enriched using an oligonucleotide mix of (ATT)_8,_ (GAGT)_2_, (CA)_10_, (GA)_10_, (GATA)_10_, (CAC)_7_, and (AGC)_7_, following previously reported protocol^56^. In brief, DNA was digested with *Rsa*I and *Xmn*I restriction enzymes, ligated to double stranded superSNX linkers, hybridized with biotinylated microsatellite oligonucleotides and captured on streptavidin coated magnetic beads. Unhybridized DNA was washed away, and captured DNA was recovered by polymerase chain reaction (PCR) using the single stranded superSNX-F as primer. The PCR products were ligated into the pCR^®^4-TOPO^®^ TA vector (Invitrogen) and transformed into the XL1-Blue competent cells. Colony PCR was performed to select the amplicons of size 500–1000 bp. In total, 155 of the 240 colonies screened contained inserts of size more than 500 bp, which were sequenced using M13 forward and reverse primers, on ABI Prism 3100 Genetic Analyzer (Applied Biosystems). Among these, 46 had non-redundant microsatellite repeats, out of which a majority were found to harbor dinucleotide repeat motifs (65.2%), followed by tri- (17.4%), tetra- (6.5%) and complex (10.9%) repeat motifs. Among dinucleotide repeat motifs CA was found to be more abundant (60%) followed by GA (30%) and AT (10%). No CG motif was observed. A total of 16 clones were eliminated because the microsatellite sequences did not have sufficient flanking regions and so primer sequences could not be designed for amplification. Therefore, primers were designed for the rest of the 30 genomic microsatellite loci. Out of which, eight loci either did not yield expected product size or produced multiple non-specific bands. After the initial PCR assays, 22 microsatellite loci could be successfully amplified.

### EST SSRs

*A. mylitta* unigene EST sequences^57^ were downloaded from WildSilkBase. Out of 720 non-redundant EST sequences 50 were found to contain microsatellite repeats and 11 loci had insufficient flanking sequences to design the primer pairs. A total of 14 loci were selected based on the length and type of repeat motifs (di, tri, tetra and penta) for primer synthesis.

### PCR amplification and genotyping of SSRs

We used Primer3 program^58^ to design primers flanking SSRs in the EST clusters and microsatellite enriched sequences. PCR was carried out in a Mastercycler Gradient (Eppendorf, Germany) in a 10 μl reaction containing 1X PCR buffer (MBI Fermentas), 100 μM dNTPs, 1.0 to 3.0 mM MgCl_2_, 5 pmole of each primers, 10 ng of genomic DNA as template and 0.5 U *Taq* polymerase (MBI Fermentas). The PCR conditions were initially at 94 °C for 3 minutes as initial denaturation and 35 cycles of: 94 °C denaturation for 30 seconds, appropriate annealing temperature (established empirically) for 30 seconds and 72 °C extension for 45 seconds and 72 °C for 10 minutes as final extension. Of the 14 EST-SSRs and 30 genomic SSRs, 10 and 22 respectively, gave amplification of exact size (Remaining were with multiple bands) as assessed by 1.5% agarose gel electrophoresis (Supplementary Table S1).

To identify microsatellite loci that are polymorphic, we randomly selected two individuals from each of the eight ecoraces (Fig. 1 and Table 1) and screened all the 32 microsatellite markers (Table 1 in Supplementary file). Out of these, we finally selected 10 microsatellite marker loci based on polymorphism and absence of non-specific PCR amplifications. In all, 154 individuals from the eight ecoraces were genotyped using 10 microsatellite markers. For all the amplifications we used FAM-labeled fluorescent forward primer and unlabeled reverse primer. The PCR products were run on an ABI PRISM 3730 DNA analyzer, with Pop-7 as sieving matrix, HiDi-Formamide as single-stranded DNA stabilizer and GeneScan 500 ROX as a size standard. Subsequently, ABI PRISM GeneMapper software version 3.0 was used to size the alleles. Data on allele sizes in each individual was tabulated for genetic analysis.

### Statistical analysis

#### Descriptive statistics and Hardy-Weinberg equilibrium

For each locus, number of alleles (*A*), number of alleles in each polymorphic locus (*Ap*) (polymorphism cut-off = 99%), expected heterozygosity as Nei’s unbiased estimates of genetic diversity (H_*E*_)^59^, observed heterozygosity (H_*O*_) and Weir and Cockerham’s^60^ estimate of inbreeding coefficient (f) were assessed using Genetic Data Analysis 1.0^61^. Exact test for Hardy-Weinberg equilibrium (HWE) with the explicit alternative hypothesis of heterozygote deficiency^62^,^63^ was performed for each locus in each population using GENEPOP 4.0.5.3^64^,^65^. Locus specific global tests taking all the populations together were also performed with the same software. All tests were performed with the default parameters of 1000 dememorization steps and 20 batches per locus, with 5000 iterations per batch. All inferences were drawn following sequential Bonferroni correction^66^. The probability of null allele occurrence was estimated using MICROCHECKER^67^ in which null alleles were considered to occur at a locus if an overall significant excess of homozygotes is seen, distributed evenly across the homozygote classes.

#### Linkage disequilibrium

Exact test for genotypic composite linkage disequilibrium (LD)^68^ for each locus pair in each population was performed in GENEPOP with the default settings of 1000 dememorization steps, 20 batches per test and 5000 iterations per batch^64^,^65^. The null hypothesis of an equilibrium was accepted or rejected following sequential Bonferroni correction.

#### Population differentiation and Mantel test

Pairwise F_ST_ values and permutation test to estimate their significance was performed in ARLEQUIN 3.5 using 10,000 permutations. Pairwise physical distance in kilometers using the GPS coordinates of the sampling sites was estimated using GEOGRAPHIC DISTANCE MATRIX GENERATOR^69^. Correlation between Slatkin’s linearized estimate of F_ST_^70^ and genetic distance, i.e. isolation by distance (IBD), was tested with Mantel test^71^ in Mantel Tester, which is the program zt, with a user friendly GUI^72^. Mantel test was inferred following 10,000 permutations. In addition, a Mantel test was done between the absolute residuals of a regression of F_ST_/(1- F_ST_) upon physical distance (km), and physical distance, to check for the existence of migration-drift equilibrium, one of the basic assumptions of IBD^48^,^73^.

#### Bottleneck analysis

The BOTTLENECK program^74^ was used to determine if any signal of past bottleneck could be detected. The program was run under infinite alleles model (IAM) and two-phase mutation model (TPM)^75^ with probabilities of single step and multi step mutations to be 0.9 and 0.1 respectively^76^. When a population undergoes reduction in population size it consequently loses its genetic diversity. This is reflected in both loss in number of alleles as well as observed gene diversity. In a population that has experienced such a reduction recently, the former parameter is lost faster than the latter. Therefore in such a population one would expect observed gene diversity to be higher than expected gene diversity estimated from the number of alleles (k)^77^,^78^. BOTTLENECK is equipped with three different statistical methods to test for this, i.e., i) sign test, ii) standardized difference test and iii) Wilcoxon sign-rank test. The first suffers from very low statistical power, the second can only be performed if the number of loci is more than 20, the third is recommended for mot situations where there are reasonable numbers of markers and samples. Therefore Wilcoxon sign-rank test was used to compare the simulated gene diversity with the observed gene diversity.

#### Analysis of MOlecular Variance

Hierarchical population structure was evaluated with the AMOVA^60^,^68^,^79^ using the software ARLEQUIN 3.0. The analysis was performed choosing two main hierarchical structures; 1) Taking all the eight populations together and including the individual level and 2) Taking the Andhra Local and Bhandara populations together in one group and the rest six populations in another group (Rationale for doing so is in the ‘Discussion’ section). As the frequency of missing data was very low (maximum frequency of missing data was 3% for Amysat025), both haplotype and locus by locus analyses were performed and their results compared. “Pairwise differences” was chosen as the distance method and 10,000 permutations were used to test for the significance of covariance components and fixation indices. ARLEQUIN also returned bootstrap percentile values for the fixation indices. More than 20,000 bootstraps were carried out for each fixation index.

#### Population analysis using distance methods

There are several methods of estimating distances among populations from microsatellite allele frequencies and construct a tree from it. There are traditional distance estimates such as Nei’s standard genetic distance D_ST_^80^, Cavalli-Sforza and Edward’s chord distance D_C_^81^ etc., which are designed for loci mutating following IAM. Along with these, there are measures of genetic distance that have been explicitly defined to estimate distances based on microsatellite data, such as Goldstein *et al.*’s δμ^81^,^82^ and Shriver *et al.*’s D_SW_^83^. All these distance measures have their own advantages and disadvantages. However, it has been shown using both computer simulation^84^ as well as with real data^85^ that for constructing phylogenetic trees, the efficiency of (δμ)^2^ or D_SW_, is not very good. Rather, it was shown that the probability of the traditional distance methods in returning the correct tree topology is more. In these studies, the measure D_A_^86^ was found to perform better than the traditional measures as well as the microsatellite specific measures. And it was found to be efficient with both classical markers as well as microsatellites. Therefore, D_A_ was selected as the distance measure to construct a dendrogram for the microsatellite data of these eight populations. Neighbour joining (NJ) tree with 10000 bootstrap resampling of the loci^87^ was constructed using POPTREE2^88^.

#### Bayesian analysis of probable population clusters

Phylogenetic clustering method using individual genotype data although widely used suffers from several limitations. The actual tree topology is dependent on the genetic distance used and obtaining a reliable confidence interval with a few number of loci is not easy^89^. Therefore, we examined the number of distinct groups into which individuals from these eight populations can be clustered using the Bayesian clustering program STRUCTURE 2.3.1^89^,^90^,^91^. STRUCTURE uses a model based clustering technique to estimate the number of potential genetic clusters into which the individuals in a sample can be grouped. Given a series of potential clusters (K), STRUCTURE estimates the likelihood of each K to the data, which is achieved by estimating the number of groups that are in HWE and linkage equilibrium (LE). The K at which the likelihood reaches a plateau is taken as the number of optimal cluster number for the dataset. STRUCTURE was run assuming admixture and correlated allele frequency models. The populations were assumed to be in LE. The sampling locations were taken as putative population origin for the individuals. For the MCMC runs 100000 burn in period, followed by 100000 replications were used as recommended in the STRUCTURE manual. Value of K was taken from 1 to 15 and for each K, 20 iterations were performed. The most probable K was inferred from the modal value of rate of change of the LnP(D) value between successive runs (ΔK) as suggested by Evanno *et al.*^92^. This “Evanno method” has been implemented in the web based program STRUCTURE HARVESTER and it was used to estimate ΔK for each value of K and identify the mode^93^.

#### Artificial neural networks

We also analysed the data using unsupervised neural networks in the form self-organizing maps (SOM) or Kohonen maps^94^,^95^. This method allows one to explore and visualize a multidimensional dataset into a two dimensional grid. It also allows one to look for possible clusters in the data^96^. It has been shown in a recent study that under moderate differentiation, like in case of our data, classical clustering techniques like NJ, are not perfect in finding the correct number of clusters in the data. In such cases, Bayesian clustering techniques like STRUCTURE and SOM were more efficient^49^. SOM has been used extensively in ecology^97^,^98^,^99^,^100^,^101^,^102^,^103^, but there have been very few instances of its application to genetic data^44^,^53^,^104^.

The network consists of two layers, -i) the input layer containing as many nodes as there are markers in a dataset, each of which is connected to all the individual genotypes, and ii) the output layer, which contains the neurons arranged in a two dimensional grid of hexagonal lattice. Each of these neurons is represented by weight vectors (also known as the codebook vector), whose dimension is same as the number of nodes in the input.

During the training of the map, distance between each input vector and all the weight vectors are calculated, and the hexagon closest to the input vector is called its best matching unit (BMU). Following this, all the weight vectors are update so that the BMU moves closer to the input vector in the input layer. The topological neighbours of this neuron are also dragged along. This updating is continued in iterations. At the end of this process, individuals with similar multilocus genotypes will be placed closer on the SOM. This then can be visualized using different techniques. Detailed account of the theoretical background as well as the algorithm to make these maps can be found elsewhere^95^,^104^,^105^.

Our data consisted of 131 different alleles across the 10 loci, which formed the number of nodes in our input layer. For each of these 131 markers an individual was coded as 1, 0.5, or 0, if it was either homozygote, heterozygote or lacks this marker respectively^103^. Individuals who had missing data for a particular locus were coded as 0 for all alleles in that locus. We used a linear initialization of the SOM and trained it using a batch algorithm. The training was done in two phases–i) rough training phase with large neighbourhood radii and ii) fine-tuning phase with smaller radii. We further subjected this SOM to hierarchical cluster analyses using an agglomerative clustering method^106^. These analyses were performed using the SOM toolbox for MATLAB^107^ and SOMVIS^108^.

## Additional Information

**How to cite this article**: Chakraborty, S. *et al.* Genetic analysis of Indian tasar silkmoth (*Antheraea mylitta*) populations. *Sci. Rep.* **5**, 15728; doi: 10.1038/srep15728 (2015).

## Supplementary Material

Supplementary Information

Supplementary Data 1

Supplementary Data 2

Supplementary Data 3

Supplementary Data 4

## Figures and Tables

**Table 1:** *A. mylitta* ecoraces analyzed in this study.

Serial No.	Ecotype	Collection site	State	Sampling coordinates	Primary food plant	Cocoon weight (g) (Mean ± SD)	Cocoon shell weight (g) (Mean ± SD)	Silk ratio (%) (Mean ± SD)	Average filament length (m) (Mean ± SD)
1	Andhra Local	Adilabad	Andhra Pradesh	19.66N/78.53E	*Terminalia. arjuna T. tomentosa*	9.45 ± 0.52	1.57 ± 0.714	16.61 ± 1.58	700 ± 36.62
2	Bandhara	Bhandara	Maharashtra	21.09N/79.42E	*T. arjuna T. tomentosa*	6.93 ± 0.61	1.24 ± 0.74	17.89 ± 1.39	613 ± 51.39
3	Raily	Jagdalpur	Chhattisgarh	19.08N/82.02E	*Shorea robusta*	12.77 ± 1.05	2.66 ± 0.79	20.83 ± 1.08	1232 ± 36.62
4	Modal	Keonjhar	Orissa	21.63N/85.58E	*S. robusta*	14.17 ± 0.88	3.64 ± 0.77	25.68 ± 0.68	1383 ± 66.36
5	Sarihana	Santhal Pargana	Jharkhand	24.26N/87.24E	*T. arjuna T. tomentosa*	9.75 ± 0.46	1.88 ± 0.749	19.28 ± 0.45	785 ± 47.05
6	Sukinda	Sukindagarh	Orissa	21.20N/85.52E	*T. arjuna T. tomentosa*	13 ± 0.84	1.80 ± 0.49	13.58 ± 0.75	845 ± 60.33
7	Daba Bivoltine	Singhbhum	Jharkhand	23.23N/85.23E	*T. arjuna T. tomentosa*	12.10 ± 0.86	2.80 ± 0.76	12.64 ± 0.99	750 ± 51.74
8	Daba Trivoltine	Singhbhum	Jharkhand	23.23N/85.23E	*T. tomentosa*	11.06 ± 0.54	1.28 ± 0.43	11.57 ± 0.68	700 ± 48.22

**Table 2:** Microsatellite loci, primer sequences, allelic size range and F-statistics of 10 polymorphic loci in *A. mylitta*. Significant fixation indices are indicated by*.

Locus	Primer sequence	Allelic size range	F_IS_	F_ST_	F_IT_
**Amysat001**	F-5′CGTACGTACTTTTGATGGATT 3′	161–231	0.256*	0.040*	0.286*
	R-5′AAGCGACATACCTGCGTT 3′				
**Amysat013**	F-5′TCACCACATGTCACTGACTGAA 3′	192–195	1*	0.315*	1*
	R-5′TCACTACAATCAGGCGCAAT 3′				
**Amysat015**	F-5′TACCAACAGACAGCCCTCCT3′	179–199	0.811*	0.204*	0.850*
	R-5′TCAAGGCTTTGACGTTGTATG3′				
**Amysat019**	F-5′CGCCATCTTGTTAGTTCTTCG 3′	241–264	0.632*	0.213*	0.711*
	R-5′TCGGGTGCTTTCCAAAGATA 3′				
**Amysat021**	F-5′CAAGATCGCGTTATCCTTTTT 3′	183–189	0.705*	0.196*	0.763*
	R-5′TGTTGTGAAGAACCCCCATT 3′				
**Amysat023**	F-5′TGCCCAGATAGTGTTTACCG 3′	106–175	0.088*	0.041*	0.125*
	R-5′GACCGGTCTGAACATAGTTGC 3′				
**Amysat025**	F-5′GTCTGGGGAAGCTGTTAAGAC 3′	228–250	0.739*	0.094*	0.763*
	R-5′CAATCGATATTTGAAACCGCTAA 3′				
**Amysat026**	F-5′CGCCACTTGCTCAGGAAT 3′	131–157	0.029	0.017	0.045
	R-5′CACGAGATGAGGTTGTGGTG 3′				
**Amysat032**	F-5′ACACCCCAAAATGTCAATG 3′	119–200	0.588*	0.024	0.597*
	R-5′ACCGGCACCATCACAT 3′				
**Amysat037**	F-5′CGCACGCGCACACT 3′	68–79	0.415*	0.576*	0.752*
	R-5′CCTATACACAAGCGAGTAGT 3′				

**Table 3:** Result for locus by locus Global AMOVA as a weighted average over all loci.

Source of variation	Sum of squares	Variance component	Percentage variation	Fixation index	Bootstrap CI (95%)
Among populations	177.13	0.55	15.41	F_ST_ = 0.154*	(0.078–0.251)
Among individuals within populations	652.32	1.52	42.68	F_IS_ = 0.505*	(0.299–0.708)
Within individuals	227	1.49	41.91	F_IT_ = 0.581*	(0.376–0.769)
Total	1056.45	3.55			

The estimates of the respective fixation indices and their bootstrap intervals are also shown. Significant estimates are indicated by*.
