# Supplementary material for: Genetic analysis of Indian tasar silkmoth (Antheraea mylitta) populations
Source: Sci Rep. 2015 Oct 29;5:15728. doi: 10.1038/srep15728 (PMC4625160; doi:10.1038/srep15728)
Supplement: Supplementary Information [file srep15728-s1.pdf]

Genetic analysis of Indian tasar silkmoth (*Antheraea mylitta*) populations

Saikat Chakraborty[1, 2#], M Muthulakshmi[1#], Deena Vardhini[1], P Jayaprakash[3], J Nagaraju[1,4] and K. P. Arunkumar[1*]

[1] Centre of Excellence for Genetics and Genomics of Silkmoths, Laboratory of Molecular Genetics, Centre for DNA Fingerprinting and Diagnostics, Tuljaguda, Nampally, Hyderabad 500001, INDIA

[2] Centre for Ecological Sciences, Indian Institute of Science, Bangalore 560012, INDIA

[3] Muga silkworm seed organization and Eri silkworm seed organization, Central Silk Board, Guwahati 781006, INDIA

[4] Deceased

Table S1: Primer details of microsatellite markers developed in this study. The allele sizes were estimated by sequencing the clones.

Serila No.	Locus	Primer sequence	Microsatellite motif	Source	Annealing temperature (°C)	Mgcl2 (mM)	Allele size (bp)	Degree of polymorphism
1	Amysat001	F-5'CGTACGTACTTTTGATGGATT 3'<br>R-5'AAGCGACATACCTGCGTT 3'	$(TGCG)_3$ Nn $(TGCG)_3$ Nn $(TGCG)_2$	Genomic	57	1.5	211	Polymorphic
2	Amysat002	F-5'ATTGTAATCATAATCCTATCG 3'<br>R-5'CCGAAACAACCTACATTCC 3'	$(TA)_2$ Nn $(TA)_2$ Nn $(TA)_3$	Genomic	52	2.0	221	Monomorphic
3	Amysat003	F-5'AGCGGAGCAAAGATGAAA 3'<br>R-5'GGCAACGCAACGTTAGATA 3'	$(TA)_3$ Nn $(TA)_2$ Nn $(TA)_3$ Nn $(TA)_4$	Genomic	56	1.5	400	Monomorphic
4	Amysat004	F-5'GAATAGCCCTGCGTGGAC 3'<br>R-5'TGAAAACAACTATCACGTAAA 3'	$(TCA)_4$	Genomic	57	1.5	277	Monomorphic
5	Amysat005	F-5'CTGCCCCCTTCTGTCAG 3'<br>R-5'CTCACTACACGGGTTCCC 3'	$(CTTT)_4$	Genomic	65	1.5	200	Monomorphic
6	Amysat006	F-5'GATCTCGTCGGACGAGG 3'<br>R-5'GATCGCGCTTGCGGTT 3'	$(CA)_{12}$ Nn $(CA)_2$ Nn $(CA)_4$	Genomic	52	2.0	163	Monomorphic
7	Amysat007	F-5'CACAACATGTAAATCGGAAAG 3'<br>R-5'TCGCAAAGATGTGGGTATCA 3'	$(GT)_2$ Nn $(GT)_5$ Nn $(GT)_2$G $(GT)_3$	Genomic	52	2.0	168	Monomorphic

8	Amysat009	F-5'TAACGGAAAAGCCCAGAGTG 3'<br>R-5'CCATGGCTTATGGTTTTTGC 3'	$(AG)_5$ Nn $(AG)_6$ Nn $(AG)_5$	EST	55	1.5	219	Monomorphic
9	Amysat010	F-5'CAGACAGACAGACGGAGTGC 3'<br>R-5'AACCGAGCAGCAAACCCTAT 3'	$(AT)_5$	EST	57	2.0	187	Monomorphic
10	Amysat013	F-5'TCACCACATGTCACTGACTGAA 3'<br>R-5'TCACTACAATCAGGCGCAAT 3'	$(GT)_5$	EST	55	1.5	194	Polymorphic
11	Amysat014	F-5'CGACAGCACTTACAGCGACT 3'<br>R-5'GGACGAACTCAAGGCTGTTC 3'	$(TCT)_6$	EST	60	1.5	194	Monomorphic
12	Amysat015	F-5'TACCAACAGACAGCCCTCCT 3'<br>R-5'TCAAGGCTTTGACGTTGTATG 3'	$(TGA)_6$	EST	57	1.5	196	Polymorphic
13	Amysat018	F-5'TGCTGAAATCAAATAAAGTGTTCA 3'<br>R-5'AAAATCCCGCCAGAAACTCT 3'	$(CT)_5$	EST	55	1.5	217	Monomorphic
14	Amysat019	F-5'CGCCATCTTGTTAGTTCTTCG 3'<br>R-5'TCGGGTGCTTTCCAAAGATA 3'	$(TA)_5$	EST	55	1.5	248	Polymorphic
15	Amysat020	F-5'GATTGGCCAAATCGTTTGAT 3'<br>R-5'CACCTGGGATATAACAGTGATAAAGA 3'	$(TAAT)_3$	EST	53	1.5	126	Monomorphic
16	Amysat021	F-5'CAAGATCGCGTTATCCTTTTT 3'<br>R-5'TGTTGTGAAGAACCCCCATT 3'	$(TAACC)_3$	EST	55	1.5	190	Polymorphic
17	Amysat022	F-5'ACCCTTGAGCTAGCTGCCTA 3'<br>R-5'TCAGACAAAAGGATACAGGTGGT 3'	$(AAGC)_3$	EST	60	1.5	195	Monomorphic
18	Amysat023	F-5'TGCCCAGATAGTGTTTACCG 3'<br>R-5'GACCGGTCTGAACATAGTTGC 3'	$(GA)_{28}$	Genomic	48	1.5	166	Polymorphic
19	Amysat024	F-5'TGGAAGATCCAACAGCATACA 3'<br>R-5'GATGAGGCACGGACACCTAC 3'	$(GT)_7$ $(GA)_{24}$	Genomic	54	3.0	239	Monomorphic
20	Amysat025	F-5'GTCTGGGGAAGCTGTTAAGAC 3'<br>R-5'CAATCGATATTTGAAACCGCTAA 3'	$(CA)_5$ Nn $(CA)_3$ Nn $(CA)_7$	Genomic	50	2.0	247	Polymorphic
21	Amysat026	F-5'CGCCACTTGCTCAGGAAT 3'<br>R-5'CACGAGATGAGGTTGTGGTG 3'	$(CCA)_2(CA)_5$ Nn $(CA)_4$	Genomic	48	1.5	142	Polymorphic
22	Amysat029	F-5'TGTGAAGTGCCATATACAGAAGG 3'<br>R-5'TCAAAAACGACGTCAAGGTG 3'	$(GT)_2$ Nn $(GT)_7$ Nn $(GT)_2$ Nn $(GT)_{21}$ Nn $(GT)_3$	Genomic	56	2.5	155	Monomorphic
23	Amysat030	F-5'ACGTGCTAGTGCA 3'<br>R-5'CATTCCACTACATCA 3'	$(CA)_9$ Nn $(CA)_{19}$	Genomic	57	2.5	250	Monomorphic

24	Amysat032	F-5'ACACCCCAAAATGTCAATG 3'<br>R-5'ACCGGCACCATCACAT 3'	$(TG)_3$ Nn $(TG)_3$ Nn $(TGA)_3$ Nn $(TG)_{12}$ Nn $(TG)_6$ Nn $(TG)_3$ Nn $(TG)_6$	Genomic	48	1.5	166	Polymorphic
25	Amysat033	F-5'GAGCCCTTGAGTATGCTGCT 3'<br>R-5'ACACGCCTCAACAACTCCTC 3'	$(CA)_{11}$	Genomic	48	1.5	159	Monomorphic
26	Amysat034	F-5'ATAGGTATGTGGGGTGAATC 3'<br>R-5'CACAACACTCGCAGTGCG 3'	$(GT)_{17}$ Nn $(GT)_5$ Nn $(GT)_2$ Nn $(GT)_{25}$ Nn $(GT)_4$ Nn $(GT)_2$ Nn $(GT)_5$ Nn $(GT)_5$	Genomic	48	2.5	202	Monomorphic
27	Amysat035	F-5'CCTTCACACATACATCCTGCAC 3'<br>R-5'TAAACCGTGACTCAAGCACAA 3'	$(CA)_{23}$	Genomic	58	2.5	100	Monomorphic
28	Amysat036	F-5'TGAACGATTAAAATTGCATTTAGG 3'<br>R-5'CGTGGGGTTCTACCACCTAC 3'	$(GT)_3$ Nn $(GT)_5$	Genomic	57	1.0	227	Monomorphic
29	Amysat037	F-5'CGCACGCGCACACT 3'<br>R-5'CCTATACACAAGCGAGTAGT 3'	$(CA)_{20}$	Genomic	54	1.5	122	Polymorphic
30	Amysat038	F-5'TTCAGGTCCTCCAATGTTCC 3'<br>R-5'GTTCCACCGTCGACTGCTT 3'	$(GCA)_2$ GGG (GCA) CA $(GCA_{)6}$ GCG $(GCA)_4$ CCA $(GCA)_2$	Genomic	53	2.0	153	Monomorphic
31	Amysat040	F-5'GGGCCTCGTCTCAATTCAA 3'<br>R-5'CAAATCGAAACGGTAGTTCTTG 3'	$(AAT)_3$ Nn $(AAT)_2$ Nn $(GTTT)_5$	Genomic	53	2.0	209	Monomorphic
32	Amysat041	F-5'TGGCTTTGTAAGGTTTTTACAGC 3'<br>F-5'TGGGTATTTTGATTCTATTTGTGC 3'	$(GA)_7$	Genomic	53	2.0	169	Monomorphic

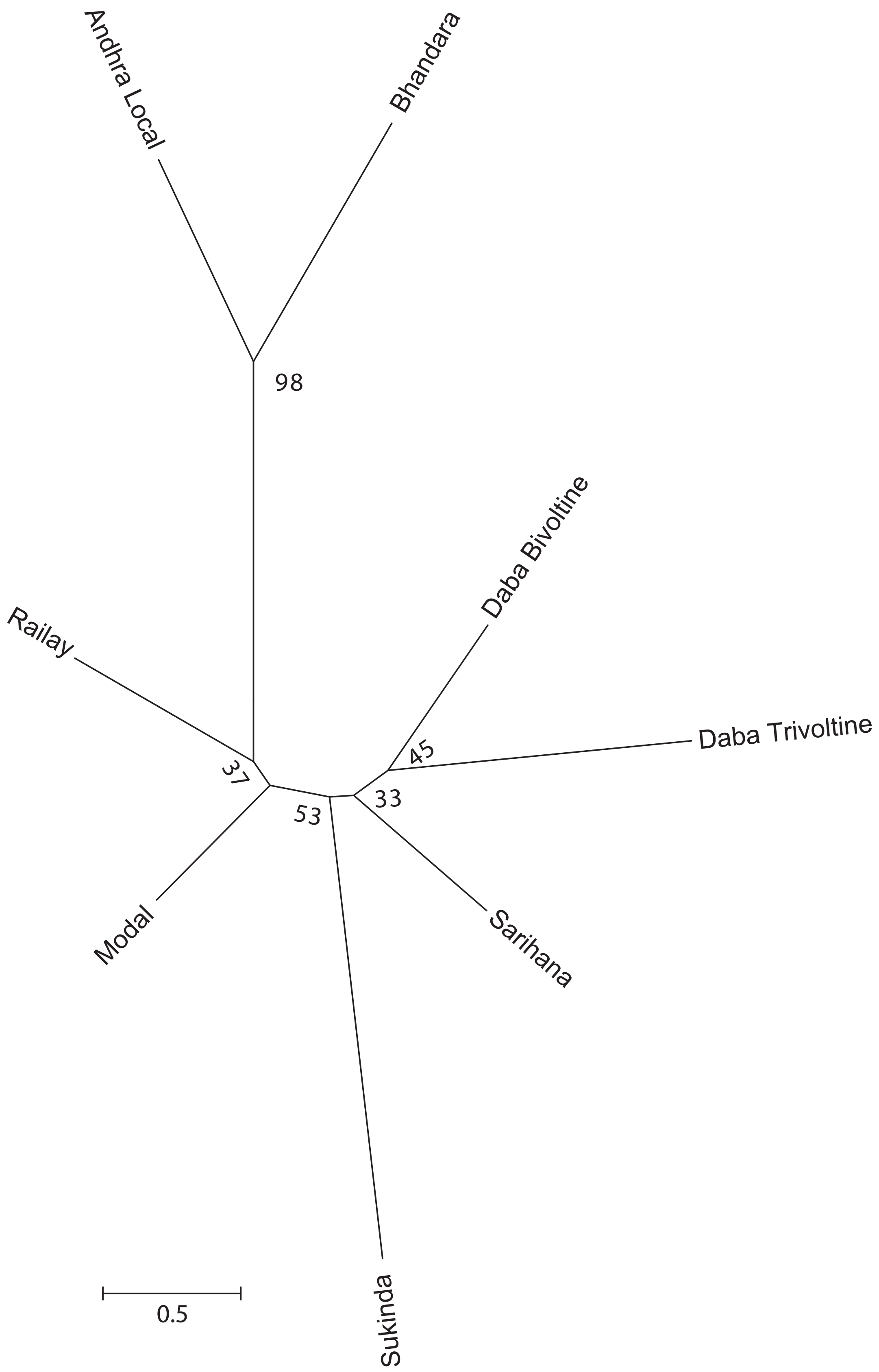

Figure S1: NJ tree constructed using Nei et al's DA. The values at the nodes represent bootstrap support

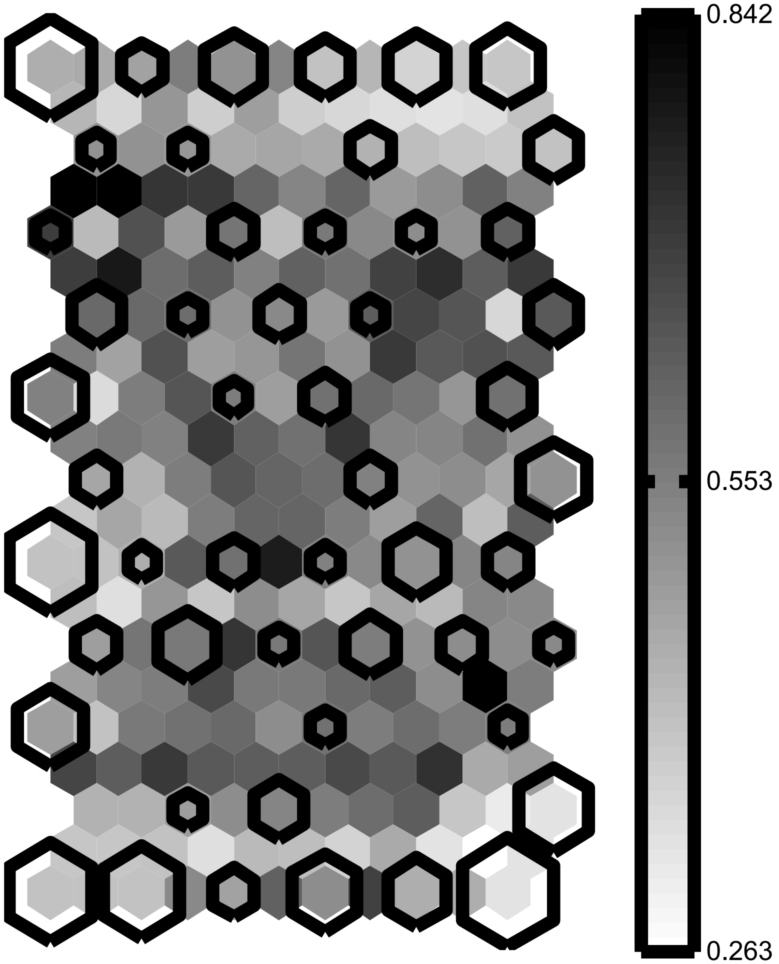

Figure S2. Hit histogram of the dataset on the U-matrix. The size of a boundary around a neuron is proportional to the number of links that neuron has with the input data (number of BMUs).

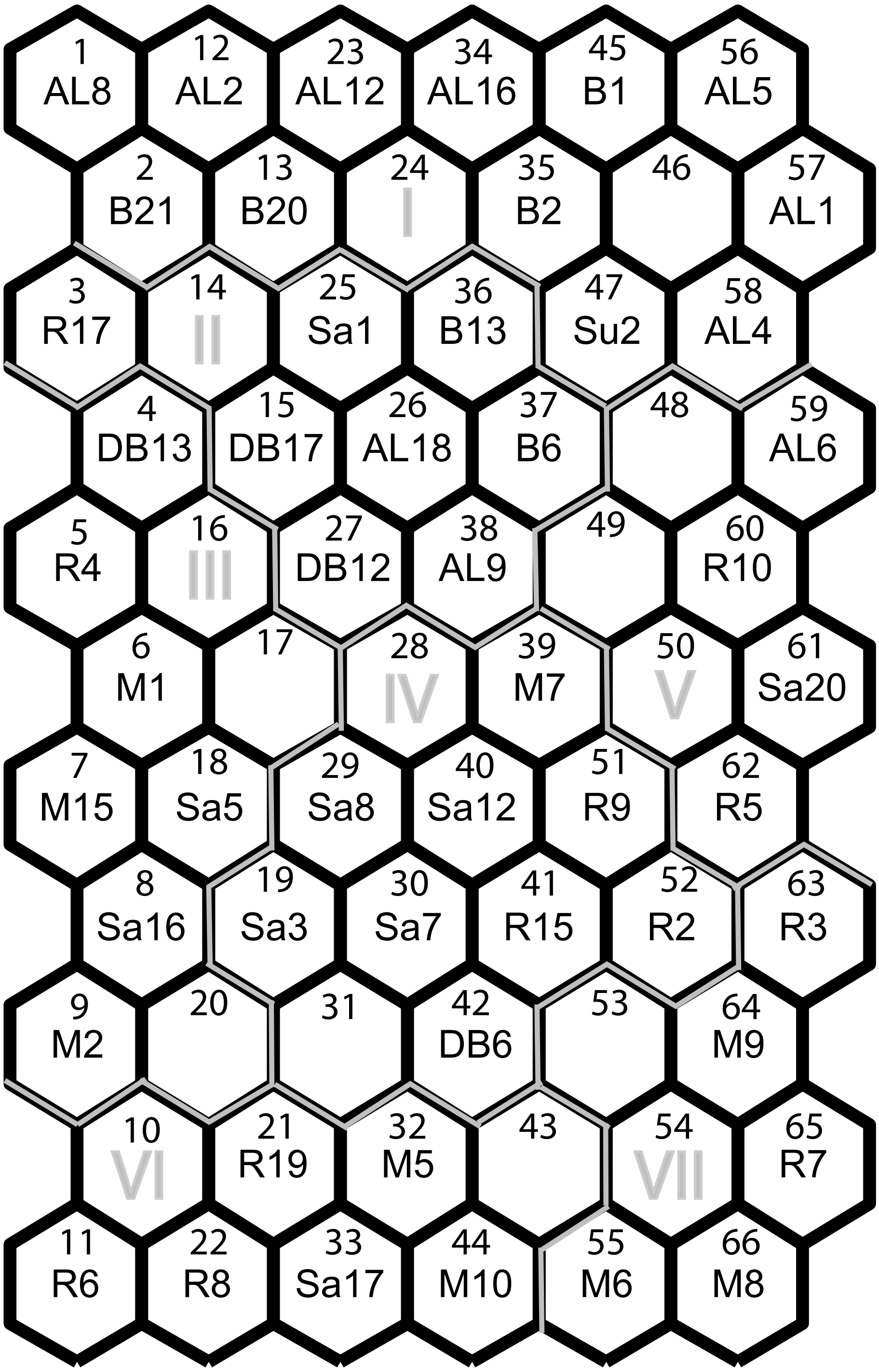

Figure S3: The self organization map from Figure 6a showing the individual labels. In addition this figure shows the clusters obtained following hierarchical cluster analysis based on Ward's linkage (Refer main text and Figure 6).

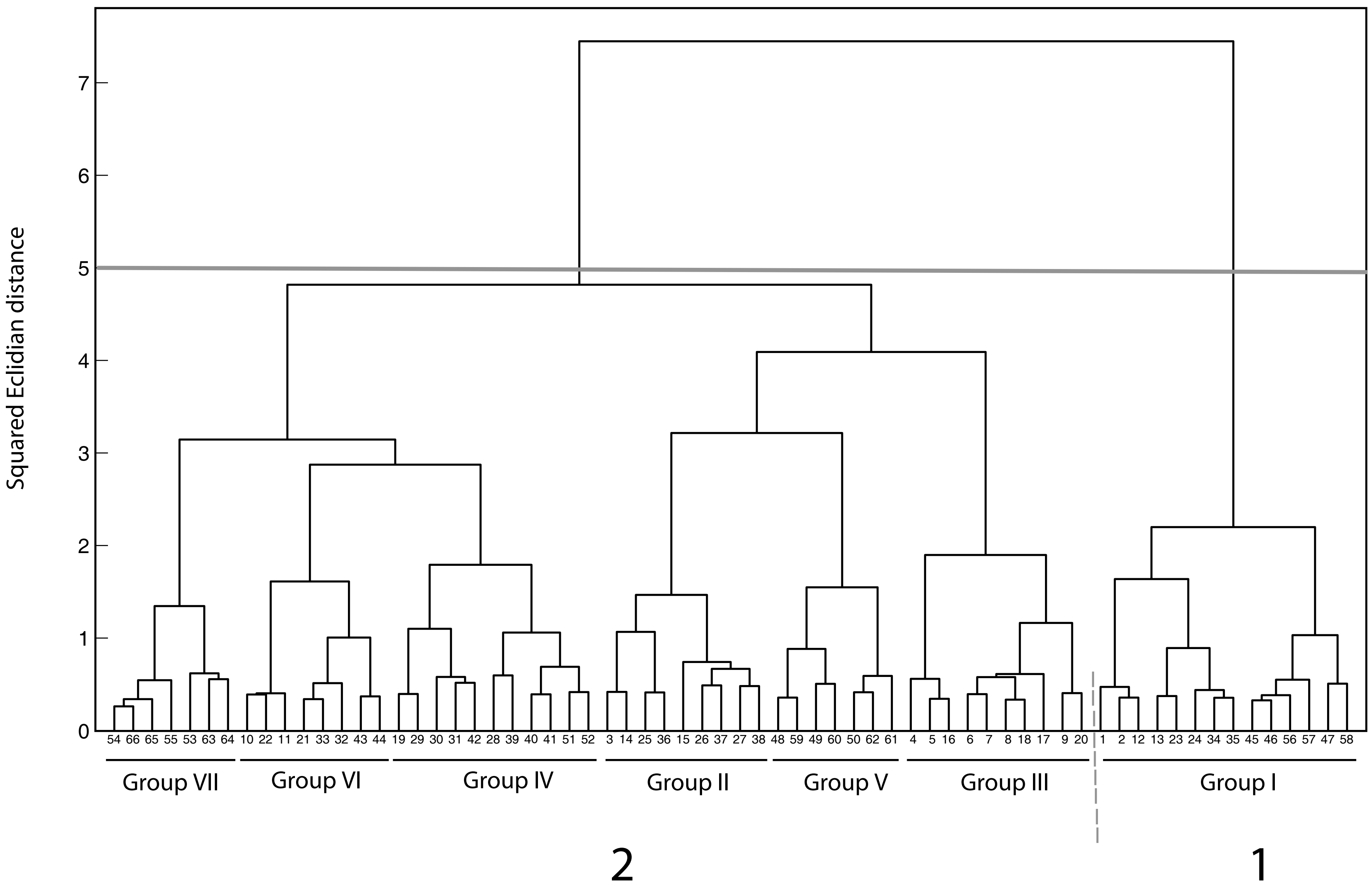

Figure S4. Dendrogram for the hierarchical cluster analysis using Ward's linkage.
